# Evaluation of reference genes in mouse preimplantation embryos for gene expression studies using real-time quantitative RT-PCR (RT-qPCR)

**DOI:** 10.1186/1756-0500-7-675

**Published:** 2014-09-25

**Authors:** Jae-Kyo Jeong, Min-Hee Kang, Sangiliyandi Gurunathan, Ssang-Goo Cho, Chankyu Park, Han Geuk Seo, Jin-Hoi Kim

**Affiliations:** Department of Animal Biotechnology, KonKuk University, Seoul, 143-701 Republic of Korea

**Keywords:** RT-PCR, Reference gene, Mouse, Preimplantation embryos, Gene expression

## Abstract

**Background:**

Real-time quantitative reverse-transcriptase polymerase chain reaction (RT-qPCR) is the most sensitive, and valuable technique for rare mRNA detection. However, the expression profiles of reference genes under different experimental conditions, such as different mouse strains, developmental stage, and culture conditions have been poorly studied.

**Results:**

mRNA stability of the *actb*, *gapdh*, *sdha*, *ablim*, *ywhaz*, *sptbn*, *h2afz*, *tgfb1*, *18 s* and *wrnip* genes was analyzed. Using the NormFinder program, the most stable genes are as follows: *h2afz* for the B6D2F-1 and C57BL/6 strains; *sptbn* for ICR; *h2afz* for KOSOM and CZB cultures of B6D2F-1 and C57BL/6 strain-derived embryos; *wrnip* for M16 culture of B6D2F-1 and C57BL/6 strain-derived embryos; *ywhaz*, *tgfb1*, *18 s*, *18 s*, *ywhaz*, and *h2afz* for zygote, 2-cell, 4-cell, 8-cell, molular, and blastocyst embryonic stages cultured in KSOM medium, respectively; *h2afz*, *wrnip*, *wrnip*, *h2afz*, *ywhaz*, and *ablim* for zygote, 2-cell, 4-cell, 8-cell, molular, and blastocyst stage embryos cultured in CZB medium, respectively; *18 s*, *h2afz*, *h2afz*, *actb*, *h2afz*, and *wrnip* for zygote, 2-cell, 4-cell, 8-cell, molular, and blastocyst stage embryos cultured in M16 medium, respectively.

**Conclusions:**

These results demonstrated that candidate reference genes for normalization of target gene expression using RT-qPCR should be selected according to mouse strains, developmental stage, and culture conditions.

**Electronic supplementary material:**

The online version of this article (doi:10.1186/1756-0500-7-675) contains supplementary material, which is available to authorized users.

## Background

The culture media used for *in vitro* propagation of mouse preimplantation embryos plays an important role in maintaining their efficacy and survival rates. Extensive efforts to establish appropriate culture conditions has led to the development of media such as the modified version of Whittingham’s original medium 16 {M16; [1]}, Chatot-Ziomek-Bavister [CZB; [2]], and Potassium Simplex Optimized Medium {KSOM; [3]} for *in vitro* culture of mouse preimplantation embryos. These culture media contain seven inorganic ions: Na^+^, K^+^, Cl^-^, Ca^2+^, Mg^2+^, SO_4_^2-^, and PO_4_^2-^ [4]: M16 was formulated in the 1970s, whereas, in the late 1980s, CZB and KSOM media were first developed in order to overcome 2-cell blocks, which are sensitive to osmolarity [5, 6]. Therefore, these media had much lower osmolalities than M16 medium, mainly due to lower inorganic ion concentrations. In contrast, the concentration of KCl, sodium lactate, sodium pyruvate, and glucose were higher in M16 than KSOM. However, M16 has a lower concentration of KH_2_PO_4_, NaCl, and antibiotics. Also, M16 has no ethylenediaminetetraacetic acid (EDTA) or L-Glutamine. EDTA was shown to affect embryonic development by inhibiting glycolysis at the two-cell stage, thereby preventing the premature stimulation of glycolysis [7, 8]. In mice, the potential for embryonic development during *in vitro* culture may differ among strains. For example, the ability of one-cell mouse embryos to develop into blastocysts *in vitro* has been shown to be a function of mouse strains, media components, and culture conditions [9–12]. In this regard, there is a need to assess the relative roles of the mouse strain background and culture environment in the modification of gene regulation during *in vitro* culture.

The scarcity of the mRNA amounts obtained from preimplantation embryos has hampered the molecular analysis of preimplantation embryos [13–15]. Recent progress in RNA amplification methods and microarray platforms, including genes unique to preimplantation embryos, allow us to apply global gene expression profiling to the study of preimplantation embryos [16–19]. Initially, the majority of studies focused on gene expression analysis of preimplantation embryos, which were based on conventional reverse-transcriptase polymerase chain reaction (RT-PCR) [20, 21]. Compared to conventional RT-PCR, several studies reported that real-time quantitative reverse-transcriptase polymerase chain reaction (RT-qPCR), in which data are accurately normalized, is significantly less variable than conventional RT-PCR procedures [22, 23]. Therefore, it is very important to establish an accurate normalization procedure to control for variability in RT-qPCR data. Generally, glyceraldehyde-3-phosphate dehydrogenase (*gapdh*), beta-actin, and ribosomal RNA are commonly used as internal control RNA. However, it has not been examined systematically whether the amount of mRNA in preimplantation embryos is variable for most genes, including reference genes, due to culture environment, media components, and mouse strains. The differences in reference gene expression among mouse strains during *in vitro* culture may introduce a considerable bias if the values of the target genes were normalized to the values of inconsistent housekeeping genes. Therefore, in order to quantify the limited quantities of mRNA contained in each embryo, a reference gene with stable expression across preimplantation embryos is required.

The expression stability and validation of reference genes suitable for the normalization of RT-qPCR data have been investigated in detail in various organisms including mouse [24–26], rabbit [27], cat [28], pig [29], bovine [22, 30–32], horse [33], and human [34, 35]. In gene expression studies on animal preimplantation embryos, normalization is generally accomplished using a single housekeeping gene. In the most recent mouse preimplantation study reported, 12 housekeeping genes were tested across *in vitro*- vs. *in vivo*-derived preimplantation embryos, and three (*ppia*, *h2afz*, and *hprt* genes) of them were used for normalization of target gene expression [25]. Although several studies have proven that the expression level of reference genes in different conditions varies, the aim of this study is to investigate the expression profiles of reference genes under different experimental conditions, such as different strain backgrounds (C57BL/6 for inbreed, B6D2F-1 for hybrids, and ICR for outbreed), culture conditions, and different development stages during the preimplantation of mouse embryos. In addition, we focused on the identification and selection of the best stable genes for normalization of gene expression analysis in different developmental stages, culture condition, and strains.

## Methods

### Animals

The mice were housed in wire cages at 22 ± 1°C under a 12 L:12D cycle with 70% humidity and fed *ad libitum*. All experiments were performed in the Institutional Animal Care and Use Committee at Konkuk University (IACUC approval number: KU12079), Seoul, Korea.

### Preparation and procurement of media

All chemicals used for media preparation were purchased from Sigma Chemical Co. (St. Louis, MO, USA). Embryo culture media, such as M16 [1], was purchased from Sigma and contained no EDTA. CZB [2] and KSOM [3] were purchased from Millipore (St. Charles, MO, USA). Amino acids purchased from Sigma were added to KSOM. Each media composition is described in Additional file 1: Table S1. All embryo manipulations outside the incubator were performed in CZB-HEPES medium (Sigma, USA).

### Embryo recovery and culture

Female ICR, B6D2F-1 and C57B/6 mice (age 6–8 wk) were superovulated by injection of 5 IU of equine chorionic gonadotropin (eCG), followed by the injection of 5 IU of hCG 48 h later, and then mated with male ICR, B6D2F-1, and C57BL/6 mice. Day 0 of gestation was defined as the day a vaginal plug was found. Plug-positive females were separated for experimentation. Zygotes were obtained by opening the ampulla at 20 h post-hCG administration using CZB-HEPES medium. Approximately 20 embryos were transferred into a 30-μL drop of fresh medium (M16, KSOM, CZB) covered with mineral oil. Embryo culture in each media was performed according to manufacturer’s protocols and using 5% CO_2_ in atmospheric oxygen at 37°C. Embryo development rates in vitro under three different culture media are shown in Additional file 2: Table S2. For recovery of in vivo-derived embryo, ICR or B6D2F-1 and C57B/6 female mice (4 to 6 wk old) were superovulated using PMSG/hCG and mated with a proven fertile male of the same strain. One cell, 2 cells, 4 cells, 8 cells, morulae and blastocyst stage embryos were recovered at 18–22, 38–42, 48–52, 64–68, 88–92 and 96–100 hrs after hCG injection, respectively.

### RNA isolation and reverse transcription

Twenty embryos were washed in Ca^+2^- and Mg^+2^-free PBS, snap-frozen in liquid nitrogen, and stored at -70°C. mRNA was extracted from groups of embryos using the Dynabeads mRNA Direct Kit (Dynal Ase) according to the manufacturer’s instructions (r = 6). For reverse transcription, total RNAs in a final volume of 20 μL (containing 0.5 mg oligo-dT, RT buffer [1×], 10 mM dithiothreitol, and 10 mM dNTP) was subjected to reverse transcription at 37°C for 50 min, followed by 70°C for 15 min, and products were stored at 4°C until use.

### Selection of reference genes and primer design

Reference genes were chosen from those used routinely in studies of pre-implantation embryonic stages [11, 36, 37]. Other potentially suitable reference genes were selected among those used in published literature on the reproductive system (Table 1) [38–41]. Whenever possible, primers fulfilled the following recommended criteria: amplicon length of 80 bp – 130 bp, location of primers on two different exons, primer sequence length of 18–25 bp, melting temperature of 58°C ± 2°C and GC content of 40% – 60%. Primer specificity was checked *in silico* (Primer-BLAST Tool from http://www.ncbi.nlm.nih.gov/tools/primer-blast/). All oligonucleotides were supplied unmodified and desalted (Cosmogenetech, Korea).

**Table 1:** **Primer sets used in this study**

Gene name	Accession number	Primer sequences (forward/reverse)	Tm(°C)	Amplication length	Amplification efficacy (%)
h2afz	NM_016750	GTGGACTGTATCTCTGTGAA/GGTTGGTTGGAAGGCTAA	60	89	92.1
sdha	NM_023281	ATTCATTGTCTACTTCTCACT/AGGGTTTATTTGGCTTACA	58	108	90.6
tgfb1	NM_011577	TATACTGAGACACCTTGG/GTGATAGTCCTGAATAATTTG	55	83	97.2
gapdh	NM_008084	AGTGGCAAAGTGGAGATT/GTGGAGTCATACTGGAACA	60	83	91.5
actb	NM_007393	ATCTTCCGCCTTAATACT/GCCTTCATACATCAAGTT	56	98	90.7
sptbn	NM_175836	TCTAATGGTTACTTGCTTGT/CAATAGTTACAGTGACAGAGA	55	101	91.2
ablim	NM_178688	GTATTCAGTGTTCACAGT/AATAGCATTAACCAGTAAGA	55	106	90.1
ywhaz	NM_001253807	CAGTAGATGGAGAAAGATTTGC/GGGACAATTAGGGAAGTAAGT	60	92	93.5
wrnip	NM_030215	ATGAGTAGGATGCTTGTA/TAACCACCTCCATCTATG	56	130	91.6
18 s	X00686	CGCCGCTAGAGGTGAAATTCT/CGAACCTCCGACTTTCGTTCT	60	102	93.6

### Real-time quantitative reverse transcriptase PCR (RT-qPCR)

The PCR reactions were performed according to the instructions of the real-time PCR machine manufacturer (ABI 7800, Applied Biosystems, Foster City, CA). The threshold cycle (Ct) value represents the cycle number at which sample fluorescence rises to a statistically significant level above the background. Each well contained 1 μL of a 10-fold dilution of cDNA, 10 μL of 2× Maxima® SYBR Green/ROX qPCR Master Mix (Thermo Scientific Fermentas, Göteborg, Sweden), 2 μL of each primer 1 –3 μM and 7 μL water. We optimized qPCR conditions on the ViiA^TM^7 real-time PCR machine according to manufacturer’s instructions and by testing different concentrations of primers and templates. The PCR program was as follows: denaturation (95°C for 10 min), amplification and quantification repeated 40 times (95°C for 10 sec, 55 – 60°C for 30 sec, and 72°C for 30 sec with a single fluorescent measurement), melting curve analysis (65 – 95°C, with a heating rate 0.2°C/sec and continuous fluorescence measurement), and final cooling to 12°C.

We confirmed the amplification of specific RT-qPCR products by performing a melting-curve step at the end of each run. Serial dilution curves for each primer allowed us to calculate RT-qPCR efficiencies. The 10-fold diluted cDNA that was used for all the amplifications was within the linear dynamic range of the calibration curve – between 1- and 1000-fold dilution. Across all the assays, none of the quantification cycle (Cq) values were higher than 44. No-template and no-reverse transcription controls were run to determine any contamination or the generation of primer dimers. All amplifications were run in triplicate, and any doubtful curves were excluded. To minimize technical variation between samples through different runs, we preferred the sample maximization method, i.e., a run contained all the samples for one gene of interest respective to one reference gene.

### Statistical analysis

GenEX qPCR data analysis software (Lotsgatan, Göteborg, Sweden) was used for implementation of quality controls and the calculation of optimal endogenous controls. This program uses the comparative Ct method for relative quantitative analysis, and the results are expressed as a fold change of expression levels. The mean value of triplicates was applied for all calculations. Medians were used to replace missing values that occurred due to inconsistencies between replicates rather than from low expression. To measure the expression stability of the candidate endogenous control genes, the commonly used program NormFinder was employed. All data are expressed as means ± SD.

## Results

### Selection of ten reference genes

For RT-qPCR analysis, total RNA was extracted from 20 zygotes or embryos. To identify the best reference genes for gene expression studies in mouse preimplantation embryos, an RT-qPCR assay based on large amounts of transcriptome data from the mammalian preimplantation embryos was designed for the transcription profiling of the ten genes (*actb*, *gapdh*, *sdha*, *ablim*, *ywhaz*, *sptbn*, *h2afz*, *tgfb1*, *18 s* and *wrnip*; Table 1). The specificity of the amplifications was confirmed by the presence of a single band of the expected size for each primer pair in agarose gels following electrophoresis, and by visualizing the single-peak melting curves of the PCR products (Additional file 3: Figure S1). The melting temperatures of all PCR products are shown in Table 1.

The cycle threshold values of candidate genes in different experimental subsets/conditions showed in Additional file 4: Table S3. All the genes tested in this study, using the NormFinder analysis, presented gene stability values (SD-value) acceptable enough for them to qualify as potential reference genes in both mouse strains and culture media. Among them, in all different experimental conditions including culture conditions, development stages and strains, the *18 s* gene had a lower average Ct value, whereas the *tgfb1* gene had a higher average Ct value. The RT-qPCR test suggested that *tgfb1* should be selected as an internal reference gene when analyzing a high-abundance target gene, while *18 s* can serve as an internal reference gene when analyzing a low-abundance target gene.

### Validation of reference genes

**Figure 1:**
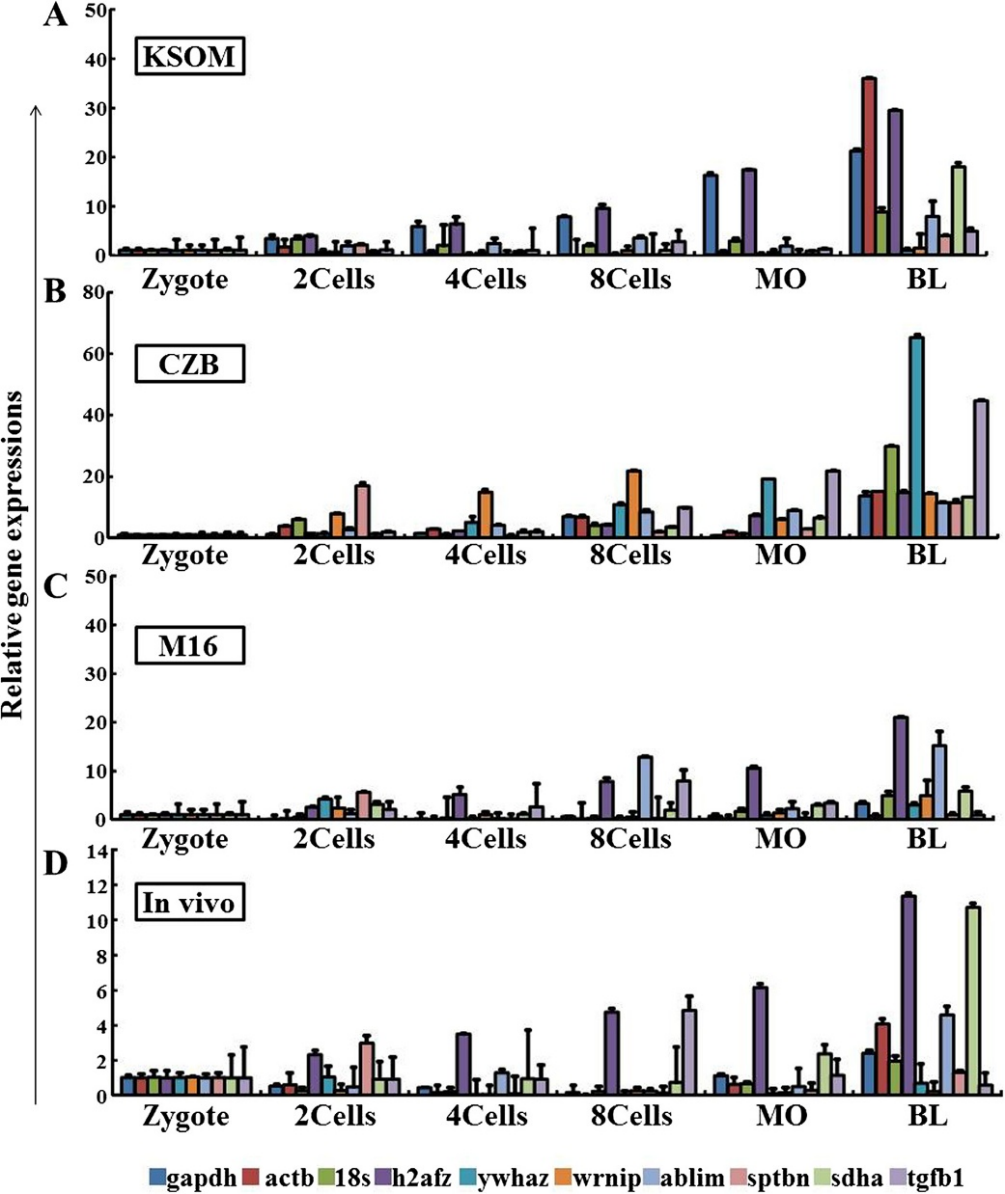
**Transcript levels of selected reference genes in the ICR mouse-derived preimplantation stage embryos.** Transcription levels of ten housekeeping genes shown for KSOM- **(A)**, CZB- **(B)**, M16- **(C)**, and *in vivo* **(D)**-derived embryos. The mRNA expression level at zygote stage was measured as a control to calculate the relative amounts in the different stages.

**Figure 2:**
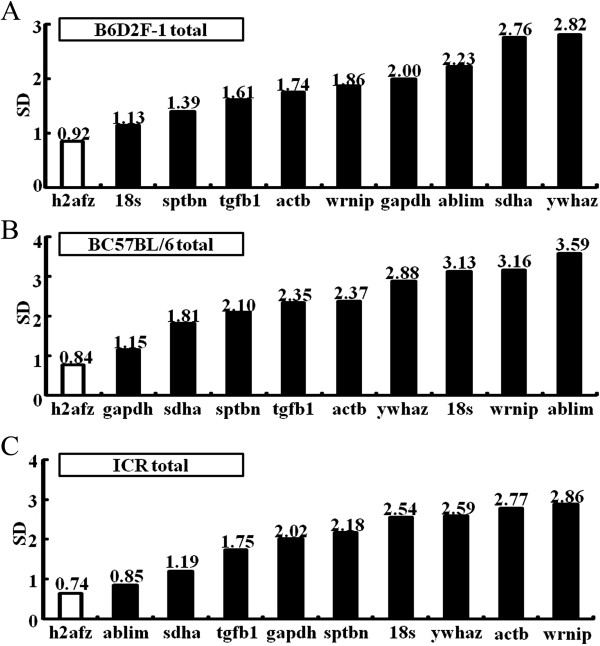
**The expression stability of reference genes in pre-implantation embryos (from zygote to blastocyst stages) derived from three different mouse strain backgrounds were analyzed by the NormFinder program.** Average gene expression stability values of reference genes are shown for B6D2F-1- **(A)**, C57BL/6- **(B)** and ICR **(C)**-derived embryos. The most stable genes are on the left and the least stable genes on the right along the X-axis. The experiments were performed in triplicate; data shown represent the mean of three independent experiments.

Gene expression during the 2-cell, 4-cell, 8-cell, morulae, and blastocyst embryonic stages were analyzed in three differently derived mouse preimplantation embryos grown under three different culture media, and in the *in vivo*-derived embryos (Figure 1; Additional file 5: Figure S2 and Additional file 6: Figure S3). The pairwise comparison of all the potential reference genes (*actb*, *gapdh*, *sdha*, *ablim*, *ywhaz*, *sptbn*, *h2afz*, *tgfb1*, *18 s* and *wrnip*) calculated using NormFinder resulted in SD-values above 1.0 for all except the *h2fz* gene, which showed 0.92 and 0.84 for the B6D2F-1 and C57BL/6 strains, respectively (Figure 2). In the ICR strains, however, both *h2afz* and *ablim* genes were below the SD-value cut-off of 1.0.

### NormFinder analysis of in Vivo vs. in vitro derived embryos

We used NormFinder analysis as a model-based approach to identify the optimal reference genes among a set of candidates. A lower average expression stability value indicates that the gene expression is more stable. NormFinder ranks all reference gene candidates based on intra- and inter-group variations and combines both results into a stability value for each reference gene candidate [42]. In B6D2F-1 embryos, *h2afz*, *actb*, and *tgfb1* are ranked as good reference genes in M16 and KSOM media, whereas *h2afz* and *18 s* are ranked as good reference genes in CZB (Table 2). Even though *actb* and *tgfb1* are the most stably expressed genes across strains cultured in M16 and KSOM media, these reference genes did not fulfill the stability criteria in CZB medium. By NormFinder analysis, the recommended comprehensive ranking of the stability of gene expression in the *in vivo*-derived embryos was determined as *h2afz* > *gapdh* > *tgfb1* > *wrnip* > *sptbn* > *18 s* > *ablim* > *ywhaz* > *sdha* > *actb*. In C57B/6 embryos, *h2afz* is the most suitable gene for studies in the three different media tested and in the *in vivo*-derived embryos. Of note, *sdha* in KSOM media is the most suitable reference gene, whereas this reference gene was identified as the least stable gene for CZM and M16 media, and *in vivo*-derived embryos (Table 2). The average gene expression stability in order of most stable to least stable genes for *in vivo*-derived embryos was determined as *h2afz* > *gapdh* > *18 s* > *wrnip* > *tgfb1* > *sptbn* > *actb* > *ablim* > *ywhaz* > *sdha*. In ICR embryos, the gene with the most stable expression for *in vitro*- and *in vivo*-derived embryos was *h2afz* (Table 2). Of note, the gene stability *of in vivo-derived* embryos contrasted with *in vitro* culture-derived embryos: most of the reference genes, except *sdha*, showed stable expression. In conclusion, the best reference gene for *in vitro*- or *in vivo*-derived embryos is *h2afz*, regardless of culture media used.

**Table 2:** **Expression stability and ranking of ten reference genes in each strains derived-embryos analyzed using NormFinder software**

Strains	Ranking	***In vitro***				***In vivo***
		KSOM	CZB	M16	Total	
B6D2F-1	1	h2afz	h2afz	wrnip	h2afz	h2afz
BC57BL/6		h2afz	h2afz	gapdh	h2afz	h2afz
ICR		gapdh	ablim	sptbn	h2afz	sptbn
B6D2F-1	2	actb	18 s	18 s	18 s	gapdh
BC57BL/6		sdha	gapdh	h2afz	gapdh	gapdh
ICR		h2afz	actb	h2afz	ablim	h2afz
B6D2F-1	3	tgfb1	sptbn	h2afz	sptbn	tgfb1
BC57BL/6		tgfb1	actb	sptbn	sdha	18 s
ICR		ablim	h2afz	ablim	sdha	ywhaz
B6D2F-1	4	ablim	gapdh	gapdh	tgfb1	wrnip
BC57BL/6		gapdh	tgfb1	actb	sptbn	wrnip
ICR		18 s	sdha	sdha	tgfb1	ablim
B6D2F-1	5	sptbn	sdha	ablim	actb	sptbn
BC57BL/6		actb	sptbn	sdha	tgfb1	tgfb1
ICR		sdha	tgfb1	tgfb1	gapdh	actb
B6D2F-1	6	18 s	wrnip	tgfb1	wrnip	18 s
BC57BL/6		sptbn	18 s	ywhaz	actb	sptbn
ICR		sptbn	gapdh	18 s	sptbn	18 s
B6D2F-1	7	wrnip	tgfb1	actb	gapdh	ablim
BC57BL/6		ablim	sdha	wrnip	ywhaz	actb
ICR		tgfb1	ywhaz	actb	18 s	tgfb1
B6D2F-1	8	ywhaz	ywhaz	sptbn	ablim	ywhaz
BC57BL/6		ywhaz	ywhaz	18 s	18 s	ablim
ICR		ywhaz	wrnip	ywhaz	ywhaz	wrnip
B6D2F-1	9	gapdh	actb	ywhaz	sdha	sdha
BC57BL/6		wrnip	wrnip	tgfb1	wrnip	ywhaz
ICR		wrnip	sptbn	gapdh	actb	gapdh
B6D2F-1	10	sdha	ablim	sdha	ywhaz	actb
BC57BL/6		18 s	ablim	ablim	ablim	sdha
ICR		actb	18 s	wrnip	wrnip	sdha

### NormFinder analysis of inbred vs. hybrid or outbred embryos

**Figure 3:**
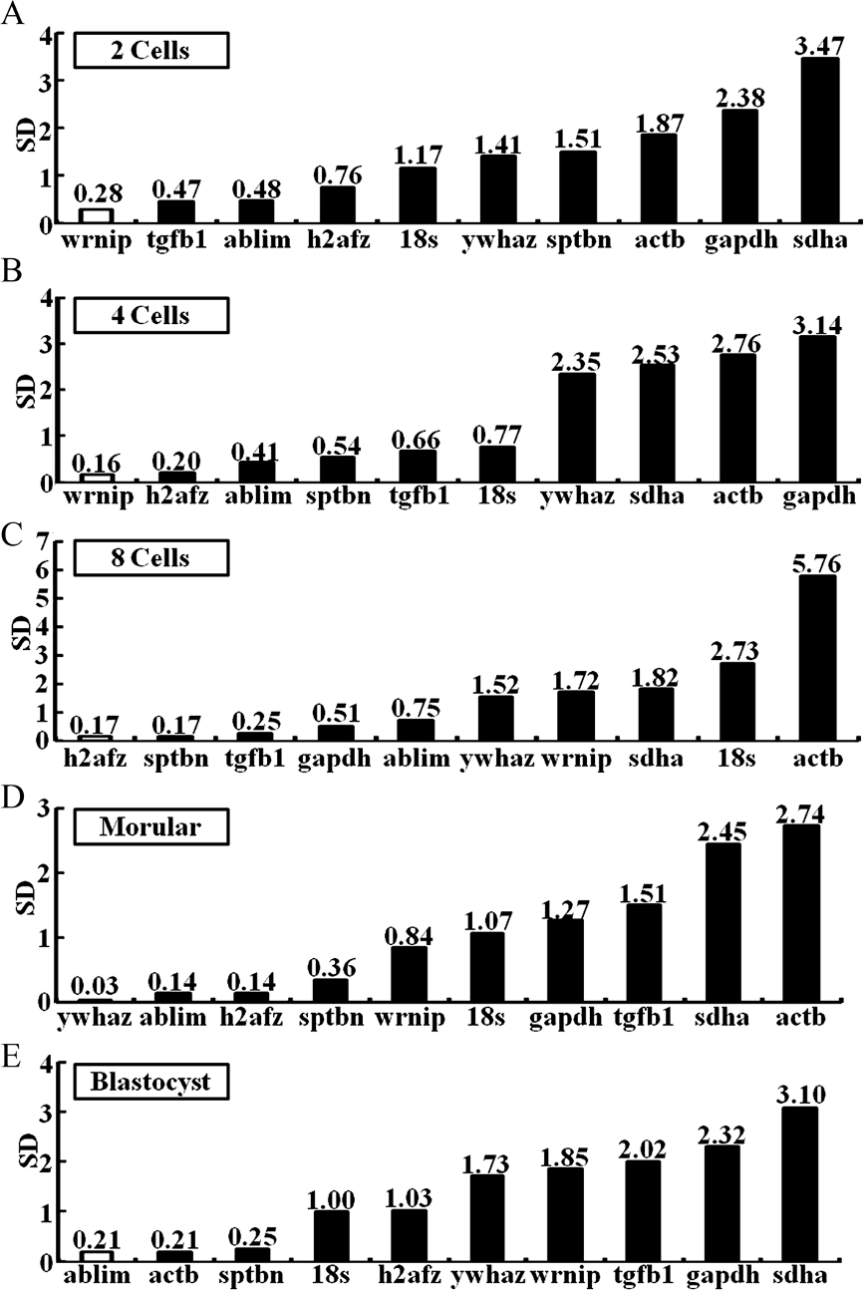
**Most stable to least stable gene expression analysis in embryos cultured in KSOM medium based on their expression stability: (A)** 2-cell, **(B)** 4- cell, **C)** 8-cell, **(D)** morulae, and **(E)** blastocyst stages. Data were obtained from **A–E**. Ranking is based on the principle that gene pairs have stable expression patterns relative to each other and are considered appropriate reference genes. The most stable genes are on the left and the least stable genes on the right along the X-axis. The experiments were performed in triplicate; data shown represent the mean of three independent experiments.

*h2afz* was found to be most stable in B6D2F-1 and C57BL/6 embryos cultured in KSOM and CZB media (Table 2), while *gapdh* was the best reference gene for analyzing B57BL/6 (Table 2) and ICR strains (Table 2) cultured in M16 and KSOM media. The wrnip gene was the most stable for evaluating the B6D2F-1 strain cultured in M16 medium (Table 2) and for the ICR strain propagated in CZB and M16 media (Table 2). The *albim* and *sptbn* genes proved to be the most stable. On the other hand, *ablim* exhibited poor stability in B6D2F-1 and B57BL/6 cultured in M16 medium, and CZB or M16 media, respectively (Figure 3B and Additional file 7: Figure S4B and C). The other genes that consistently ranked poorly included *sdha* for B6D2F-1 in KSOM and M16 media (Table 2), *18 s* for C57BL/6 and ICR cultured in KSOM and CZB, respectively (Table 2). Also, the *actb* and *wrnip* genes were least stable in ICR strain embryos cultured in KSOM and M16 media (Table 2). Even though *gapdh* and *actb* for the ICR and B6D2F-1 strains in KSOM medium emerged as the most stably expressed, consolidated evaluation under different conditions estimated that these genes were least stable in the B6D2F-1 and ICR strains (Table 2). When evaluated across three different strains, *h2afz* (B6D2F-1, C57BL/6, ICR), *18 s* (B6D2F-1), *sptbn* (B6D2F-1), *gapdh* (C57BL/6), *ablim* (ICR), and *actb* (C57BL/6, ICR) in CZB medium are the most stable, whereas *18 s* (ICR), *sptbn* (ICR), *actb* (B6D2F-1) and *ablim* (B6D2F-1) have been excluded as good candidate reference genes. The results showed that the best-ranked reference genes differed across culture conditions or mouse strains.

### Ranking order of reference genes according to developmental stages of preimplantation embryos

#### At the 2-cell stage

the ranking order of reference genes in KSOM medium are *wrnip* > *tgfb1* > *ablim* > *h2afz* > *18 s* > *ywhaz* > *sptbn* > *actb* > *gapdh* > *sdha* (Figure 3A); *h2afz* > *actb* > *wrnip* > *sdha* > *18 s* > *sptbn* > *gapdh* > *ywhaz* > *ablim* > *tgfb1* for CZB medium (Table 3); *h2afz* > *sptbn* > *ywhaz* > *sdha* > *wrnip* > *18 s* > *tgfb1* > *gapdh* > *ablim* > *actb* for M16 medium (Table 3); *tgfb1* > *ywhaz* > *18 s* > *sptbn* > *ablim* > *h2afz* > *actb* > *sdha* > *gapdh* > *wrnip* for *in vivo* embryos (Table 3). Unlike the *in vitro* culture system, *wrnip*, *h2afz*, and *tgfb1* are the least stable among the reference genes in the *in vivo*-derived embryos. Also, when evaluated across three different strains, *sptbn* is the most stable gene in the B6D2F-1 and C57BL/6 strains; *ablim* is the least stable gene, although it is the most stable gene in the ICR strains (Table 4 and Additional file 7: Figure S4, Additional file 8: Figure S5 and Additional file 9: Figure S6).

**Table 3:** **Ranking of reference genes according to development stages of pre-implantation embryo stages**

Medium	Ranking	2C	4C	8C	Mo	Bl
KSOM	1	wrnip	wrnip	h2afz	ywhaz	ablim
CZB		h2afz	h2afz	actb	h2afz	wrnip
M16		h2afz	h2afz	ywhaz	h2afz	h2afz
in-vivo		tgfb1	18 s	18 s	ywhaz	h2afz
KSOM	2	tgfb1	h2afz	sptbn	ablim	actb
CZB		tgfb1	tgfb1	h2afz	ywhaz	h2afz
M16		sptbn	sptbn	sptbn	sptbn	tgfb1
in-vivo		ywhaz	sptbn	h2afz	h2afz	ywhaz
KSOM	3	ablim	ablim	tgfb1	h2afz	sptbn
CZB		wrnip	ywhaz	ywhaz	tgfb1	ywhaz
M16		ywhaz	sdha	sdha	sdha	sptbn
in-vivo		18 s	ywhaz	gapdh	18 s	18 s
KSOM	4	h2afz	sptbn	gapdh	sptbn	18 s
CZB		sdha	18 s	sdha	wrnip	actb
M16		sdha	ywhaz	h2afz	ywhaz	ywhaz
in-vivo		sptbn	gapdh	ywhaz	gapdh	actb
KSOM	5	18 s	tgfb1	ablim	wrnip	h2afz
CZB		18 s	sdha	18 s	actb	sptbn
M16		wrnip	tgfb1	18 s	tgfb1	gapdh
in-vivo		ablim	h2afz	tgfb1	ablim	sptbn
KSOM	6	ywhaz	18 s	ywhaz	18 s	ywhaz
CZB		sptbn	sptbn	wrnip	sdha	sdha
M16		18 s	gapdh	gapdh	gapdh	sdha
in-vivo		h2afz	actb	sptbn	sdha	gapdh
KSOM	7	sptbn	ywhaz	wrnip	gapdh	wrnip
CZB		gapdh	wrnip	sptbn	sptbn	18 s
M16		tgfb1	wrnip	tgfb1	wrnip	wrnip
in-vivo		actb	tgfb1	ablim	tgfb1	sdha
KSOM	8	actb	sdha	sdha	tgfb1	tgfb1
CZB		ywhaz	gapdh	gapdh	18 s	tgfb1
M16		gapdh	18 s	wrnip	18 s	18 s
in-vivo		sdha	sdha	sdha	sptbn	tgfb1
KSOM	9	gapdh	actb	18 s	sdha	gapdh
CZB		ablim	ablim	ablim	ablim	ablim
M16		ablim	ablim	ablim	actb	ablim
in-vivo		gapdh	ablim	wrnip	actb	ablim
KSOM	10	sdha	gapdh	actb	actb	sdha
CZB		tgfb1	tgfb1	tgfb1	gapdh	gapdh
M16		actb	actb	actb	ablim	actb
in-vivo		wrnip	wrnip	actb	wrnip	wrnip

**Table 4:** **Stability rankings of ten endogenous reference genes according to development stages of pre-implantation embryo stages in each mouse strains**

Strains	Ranking	2C	4C	8C	Mo	Bl
B6D2F-1	1	sptbn	sdha	sptbn	h2afz	h2afz
C57BL/6		sptbn	ablim	ywhaz	ablim	ywhaz
ICR		ablim	gapdh	gapdh	tgfb1	ablim
B6D2F-1	2	ywhaz	sptbn	sdha	gapdh	sptbn
C57BL/6		wrnip	sptbn	sptbn	sptbn	sptbn
ICR		sptbn	sptbn	actb	wrnip	wrnip
B6D2F-1	3	sdha	ywhaz	tgfb1	ywhaz	sdha
C57BL/6		sdha	h2afz	ablim	wrnip	ablim
ICR		ywhaz	18 s	ablim	h2afz	sptbn
B6D2F-1	4	wrnip	h2afz	ywhaz	ywhaz	sdha
C57BL/6		actb	wrnip	sdha	h2afz	gapdh
ICR		wrnip	wrnip	sdha	gapdh	gapdh
B6D2F-1	5	h2afz	tgfb1	h2afz	sptbn	gapdh
C57BL/6		h2afz	gapdh	18 s	gapdh	h2afz
ICR		gapdh	sdha	h2afz	ywhaz	18 s
B6D2F-1	6	gapdh	gapdh	18 s	sdha	tgfb1
C57BL/6		gapdh	ywhaz	gapdh	ywhaz	18 s
ICR		h2afz	h2afz	sptbn	18 s	actb
B6D2F-1	7	18 s	wrnip	gapdh	18 s	ywhaz
C57BL/6		18 s	sdha	actb	sdha	sdha
ICR		sdha	actb	ywhaz	sptbn	sdha
B6D2F-1	8	tgfb1	18 s	wrnip	wrnip	18 s
C57BL/6		ablim	actb	wrnip	actb	actb
ICR		18 s	ywhaz	wrnip	actb	h2afz
B6D2F-1	9	actb	ablim	ablim	actb	actb
C57BL/6		tgfb1	18 s	h2afz	tgfb1	wrnip
ICR		actb	tgfb1	18 s	ablim	ywhaz
B6D2F-1	10	ablim	actb	actb	ablim	ablim
C57BL/6		ywhaz	tgfb1	tgfb1	18 s	tgfb1
ICR		tgfb1	ablim	tgfb1	sdha	tgfb1

#### At the 4-cell stage

The present study identified reliable reference genes among ten candidate genes for normalization of RT-qPCR data in mouse preimplantation embryos during the 4-cell development stage: *wrnip* > *h2afz* > *ablim* > *sptbn* > *tgfb1* > *18 s* > *ywhaz* > *sdha* > *actb* > *gapdh* for KSOM (Figure 3B); *h2afz* > *actb* > *ywhaz* > *18 s* > *sdha* > *sptbn* > *wrnip* > *gapdh* > *ablim* > *tgfb1* for CZB (Table 3); *h2afz* > *sptbn* > *sdha* > *ywhaz* > *tgfb1* > *gapdh* > *wrnip* > *18 s* > *ablim* > *actb* for M16 (Table 3); *18 s* > *sptbn* > *ywhaz* > *gapdh* > *h2afz* > *actb* > *tgfb1* > *sdha* > *ablim* > *wrnip* for *in vivo*-derived embryos (Table 3). Notably, *wrnip* and *18 s* in KSOM and *in vivo-*embryos are most stable, whereas these genes in the *in vivo*- and M16-derived embryos did not show a highly stable expression pattern. Although *sdha* (KSOM and CZB), *ablim* (CZB, M16) and *gapdh* (KSOM ,CZB and M16) were the least stable genes under the different culture conditions tested, *sdha* (B6D2F-1), *albim* (C57Bl/6) and *gapdh* (ICR) displayed a stable expression pattern in the corresponding strain backgrounds (Table 4 and Additional file 7: Figure S4, Additional file 8: Figure S5 and Additional file 9: Figure S6).

#### At the 8-cell stage

Next, we identified reference genes for 8-cell stages using KSOM-, CZB-, and M16-derived embryos or *in vivo*-derived embryos. The order of gene stability amongst the 8-cell stage embryos was *h2afz* > *sptbn* > *tgfb1* > *gapdh* > *ablim* > *ywhaz* > *wrnip* > *sdha* > *18 s* > *actb* for KSOM (Table 3); *actb* > *h2afz* > *ywhaz* > *sdha* > *18 s* > *wrnip* > *sptbn* > *gapdh* > *ablim* > *tgfb1* for CZB (Figure 3C); *ywhaz* > *sptbn* > *sdha* > *h2afz* > *18 s* > *gapdh* > *tgfb1* > *wrnip* > *ablim* > *actb* for M16 (Table 3) and *18 s* > *h2afz* > *gapdh* > *ywhaz* > *tgfb1* > *sptbn* > *ablim* > *sdha* > *wrnip* > *actb* for *in vivo*-derived embryos (Table 3). Unlike the other developmental stages, there was significant discrepancy in the ranking order of reference genes under each different culture conditions. When examined across three different strains, *sptbn*, *sdah*, *tgfb1*, and *ywhaz* were the most stable in B6D2F1 strains and *ywaz* and *sptbn* had highly stable expression patterns in the C57BL/6 strain background. However, in ICR strains, *gapdh* is only stable gene (Table 4 and Additional file 7: Figure S4, Additional file 8: Figure S5 and Additional file 9: Figure S6).

#### At morulae stages

*ywhaz* > *ablim* > *h2afz* > *sptbn* > *wrnip* > *18 s* > *gapdh* > *tgfb1* > *sdha* > *actb* were found to be the most stably expressed reference genes when mouse preimplantation embryos were cultured in KSOM (Figure 3D). The order of stability was *h2afz* > *ywhaz* > *tgfb1* > *wrnip* > *actb* > *sdha* > *sptbn* > *18 s* > *ablim* > *gapdh* (Table 3) and *h2afz* > *sptbn* > *sdha* > *ywhaz* > *tgfb1* > *gapdh* > *wrnip* > *18 s* > *actb* > *ablim* (Table 3) for CZB and M16 media, respectively. For *in vivo*-derived embryos, the most to least stably expressed genes were as follows: *ywhaz* > *h2afz* > *18 s* > *gapdh* > *ablim* > *sdha* > *tgfb1* > *sptbn* > *actb* > *wrnip* (Table 3). During these stages, *ywhaz* is the most stably expressed of the reference genes, while *18 s*, *ablim* and *gapdh* were the least stable. But, the *ywhaz* gene was the least stable gene in ICR strains, although it was stably expressed in the B6D2F-1 and C57Bl/6 strains (Table 4 and Additional file 7: Figure S4, Additional file 8: Figure S5 and Additional file 9: Figure S6).

#### At the blastocyst stage

The order of the most stably expressed reference genes to the least stable genes in KSOM, CZB, and M16 media are as follows: *ablim* > *actb* > *sptbn* > *18 s* > *h2afz* > *ywhaz* > *wrnip > tgfb1* > *gapdh* > *sdha* for KSOM medium (Additional file 9: Figure S6E); *wrnip* > *h2afz* > *ywhaz* > *actb* > *sptbn* > *sdha* > *18 s* > *tgfb1* > *ablim* > *gapdh* for CZB medium (Table 3); *h2afz* > *tgfb1* > *sptbn* > *ywhaz* > *gapdh* > *sdha* > *wrnip* > *18 s* > *ablim* > *actb* for M16 medium (Table 3): *h2afz* > *ywhaz* > *18 s* > *actb* > *sptbn* > *gapdh* > *sdha* > *tgfb1* > *ablim* > *wrnip* for *in vivo* blastocyst (Table 3). During this stage, *h2afz* and *ywhaz* are the most stably expressed reference genes, whereas *wrnip* is the least stable gene. Consistent with these results, data for the B6D2F-1 and C57Bl/6 strains showed that *h2afz* and *ywhaz* are the most stably expressed, while the *wrnip* gene was the most stable in the ICR strain (Table 4 and Additional file 7: Figure S4, Additional file 8: Figure S5 and Additional file 9: Figure S6). Thus, these results showed that culture conditions and mouse strains are the main factors affecting the stable expression of reference genes in the RT-qPCR experiments.

## Discussion

RT-qPCR is an invaluable technique for investigating changes in gene expression during preimplantation embryonic stages [43–45]. Since it has be performed on limited quantities of mRNA contained in each embryo, the reliability of this method mainly depends on the use of validated, stably expressed reference genes for the normalization of mRNA expression [46]. However, to the best of our knowledge, no such study on gene expression and stability in different strains cultured under different conditions has been published. The present work was thus undertaken to emphasize the need to validate the expression stability of reference genes in preimplantation embryos using different mouse strains and varied culture conditions.

An ideal reference gene is one that is stably expressed within the samples to be compared, regardless of tissue differences, experimental conditions or treatments [47, 48]. ‘Housekeeping’ genes are often supposed to have a steady expression pattern, and have been used extensively as reference genes [49]. However, many reports have shown that the expression levels of internal standards, including some housekeeping genes such as *gapdh*, *actb*, or *18 s*, can alter considerably in response to alterations in the experimental conditions [50–52]. In this study, we also reconfirmed that several of the commonly used reference genes, including *gapdh*, 18 s rRNA, and beta-actin, are unsuitable for normalization during *in vitro* culture of some of mouse strain-derived embryos.

In this study, we selected ten genes (*actb*, *gapdh*, *sdha*, *ablim*, *ywhaz*, *sptbn*, *h2afz*, *tgfb1*, *18 s* and *wrnip*) because they play different cellular roles: *actb, ablim* and *sptbn* encode cytoskeletal components expressed in various types of cells [53–55]; *gapdh* encodes an enzyme that catalyzes glycolysis for energy and carbon molecules [56]; *ywhaz* is implicated in the protection of cells from apoptosis through binding to the pro-apoptotic protein [57]; *sdha* encodes the enzyme that catalyzes oxidation in the succinate pathway [58]; *h2afz* encodes a component of the nucleosome structure of the chromosomal fiber [59]; *18 s* encodes a part of the ribosomal RNA [60]; *tgfb1* is a multifunctional component that controls proliferation and differentiation in several cell types [61]; and *wrnip* interacts with the N-terminus of the Wener protein containing the exonuclease domain [62]. KSOM and CZB media were chosen because they were optimized for inbred and outbred strains [3]. M16 medium, on the other hand was chosen, because it had higher osmolalities than KSOM and CZB media: CZB medium differs from the M16 medium, since it contains a high lactate:pyruvate ratio, 1 mM glutamine and lacks glucose. In addition, the B6D2F-1, C57BL/6, and ICR strains were chosen because these strains are well-defined and frequently used in embryological research.

In this study, we compared the candidate genes and established a stability ranking using the NormFinder software. The stability of gene expression and, therefore, the choice of reference gene for ICR strains varied considerably based on the culture media used. The most stable reference genes for KSOM, M16, and CZB media or *in vivo* embryos are the *gapdh*, *ablim*, and *sptbn* genes, respectively (Additional file 8: Figure S5). The gene *h2afz* was the most stable reference for B6D2F-1, except that *wrnip* was more stable than *h2afz,* when cultured in M16 medium (Table 2). In the B6D2F-1 and C57BL/6 strains (Table 2), the best stable reference for CZB medium is *h2afz*, whereas *ablim* is the best suitable gene for the ICR strain (Table 2). In the mouse embryos cultured in KSOM medium, the results of NormFinder software indicate that the *h2afz* gene is the most stable reference gene in the zygote and 8-cell stages, whereas *wrnip* is the most stable reference gene in the 2-cell and 4-cell stages, *ywhaz* for the morulae stage, and *ablim* for the blastocyst stage (Figure 3). In the mouse embryos cultured in CZB medium, *18 s* is the most stable reference gene in the zygote stage, whereas *h2afz* is the most stable in the 2-cell, 4-cell and morulae stages, *actb* for the 8-cell stage, and *wrnip* for the blastocyst stage (Table 3). In M16 medium, *ywhaz* is the most stable housekeeping gene in the zygote and 8-cell stages. The *h2afz* gene is the most stable of the reference genes in the 2-cell, 4-cell, morulae, and blastocyst stages (Table 3). This difference may be caused by culture media or the difference in developmental stages. Also, the reference gene expression levels in each of the developmental stages were shown in the different mouse strains tested (Figure 1; Additional file 5: Figure S2 and Additional file 6: Figure S3). Taken together, our observed data suggested that candidate reference genes to normalize and analyze target gene expression should be selected according to mouse strain, culture conditions, and developmental stages of the embryos.

The majority of gene expression studies on preimplantation embryos have been performed using only one housekeeping gene [63–65]. Contrary to our results, Chang *et al.* [66] reported significantly lower stability values for four reference genes (*sdha*, *sptbn*, *ablim* and *wrnip*); for example, *sptbn* had a higher stability value in our experiments. The differences in expression stability may be the result of different media or developmental stages analyzed in the compared experiments. The second observed difference relates to the ranking of the stability of the reference genes; for example, *wrnip* was found to be one of the two most stable genes in a recent study, but is one of the least stable genes in our study. The regulation of reference genes is not only variety/cultivar specific, but may also be developmental stage- or strain-specific and influenced by the experimental conditions [67–69]. Some genes have a relatively constant expression level across tissues while others do not [23]. This calls for validation and selection of appropriate housekeeping genes for specific strains and various experimental conditions.

## Conclusions

In the present study, we examined 3 genetic backgrounds (ICR, C57BL/6, and B6D2F-1), 6 different developmental stages (1, 2, 4, 8-cells, morulae and blastocyst), and 4 environment factors (3 culture media and in vivo control) for mRNA stability and abundance of mouse preimplantation embryos. These required so many combinations to validate each embryo. Since we used oligo-dT to reverse transcribe the mRNAs, random hexamers in addition to oliogo-dT and/or reference-free method such as mRNA sequence for evaluation of each embryo were not checked. Therefore, the RNA extraction method, reference-free methods such as mRNA-sequence, and length of the poly-A tail, which has stability as well as regulatory functions (some mRNAs have long or short poly-A tail as part of their post-transcriptional mode of regulation) remain open. In summary, we have evaluated the expression stability of various reference genes using different culture conditions and strain backgrounds to identify suitable reference genes for normalization, and NormFinder was used to calculate the normalization factor for different rankings. The results of this study indicate that *h2afz* is a better choice than other reference genes when using a single reference gene to assess target gene expression. This study provides the first assessment of new reference genes for gene expression analysis in preimplantation mouse embryos based on culture conditions, mouse strain backgrounds, and embryonic stages. These candidates may serve as better reference genes than the traditional housekeeping genes in achieving valid and reliable analysis of gene expression.

## Electronic supplementary material

Additional file 1: Table S1: Composition of each medium used in this experiment. (XLS 22 KB)

Additional file 2: Table S2: Effects of EDTA on the development of 1 cell ICR mouse embryos in vitro. (XLS 18 KB)

Additional file 3: Figure S1: Selected reference gene specificity and amplification length. **A**. specific PCR product was analyzed on agarose gel (1.5%) electrophoresis for candidate housekeeping genes. Lane M : 100 bp DNA ladder marker. Lane 2 : *ywhaz*, Lane 3 : *sdha*, Lane 4 : *tgfb1*, Lane 5 : *gapdh*, Lane 6 : *h2afz*, Lane 7 : *wrnip*, Lane 8 : *actb*, Lane 9 : *sptbn*, Lane 10 : *ablim*, Lane 11 : *18s*. **B**. Melting curve analysis of ten reference genes showing a single peak. (JPG 158 KB)

Additional file 4: Table S3: Cycle threshold values for RT- qPCR of 10 reference genes. (XLS 26 KB)

Additional file 5: Figure S2: Selected reference gene expression levels of transcripts in the B6D2F-1 mouse-derived pre-implantation stage embryos. The transcript levels are shown for KSOM- **(A)**, CZB- **(B)**, M16- **(C)**, and *in vivo* **(D)**-derived embryos. The expression at zygote stage was measured as a reference to calculate the relative amounts in the different stages. (JPG 89 KB)

Additional file 6: Figure S3: Candidate housekeeping gene expression levels of different transcripts detected in the C57BL/6 mouse-derived pre-implantation stage embryos. The expression levels of reference gene transcripts is shown for KSOM- **(A)**, CZB- **(B)**, M16- **(C)**, and *in vivo* (D)-derived embryos. The expression at zygote stage was measured as a reference to calculate the relative amounts in the different stages. (JPG 96 KB)

Additional file 7: Figure S4: Rankings of selected housekeeping genes in *in vivo*-derived each stages embryos in B6D2F-1 mouse strains: **A)** 2-cell, **B)** 4-cell **C)** 8-cell **D)** morulae, and **E)** blastocyst stages. Data were obtained from A–E. Standard deviation (SD) of traditional housekeeping genes using NormFinder. The most stable genes are on the left and the least stable genes on the right. The experiments were performed in triplicate; data shown represent the mean of three independent experiments. (JPG 117 KB)

Additional file 8: Figure S5: Average stability rankings of ten endogenous reference genes in each stages embryos in C57BL/6 mouse strains: 2-cell, **B)** 4- cell, **C)** 8-cell, **D)** morulae, and **E)** blastocyst stages. Data were obtained from **A–E** and analyzed for the SD of endogenous reference genes using the NormFinder program. The most stable genes are on the left and the least stable genes on the right. The experiments were performed in triplicate; data shown represent the mean of three independent experiments. (JPG 116 KB)

Additional file 9: Figure S6: Rankings of ten housekeeping genes in *in vivo*-derived each stages embryos in ICR mouse strains: 2-cell, **B)** 4- cell, **C)** 8-cell, **D)** morulae, and **E)** blastocyst stages. Data were obtained from **A–E**, respectively. Ranking is based on the principle that gene pairs have stable expression patterns relative to each other and are considered appropriate housekeeping genes. The most stable genes are on the left and the least stable genes on the right. The experiments were performed in triplicate; data shown represent the mean of three independent experiments. (JPG 117 KB)
