# Supplementary figures and images for: Evaluation of reference genes in mouse preimplantation embryos for gene expression studies using real-time quantitative RT-PCR (RT-qPCR)
Source: BMC Res Notes. 2014 Sep 25;7:675. doi: 10.1186/1756-0500-7-675 (PMC4181407; doi:10.1186/1756-0500-7-675)

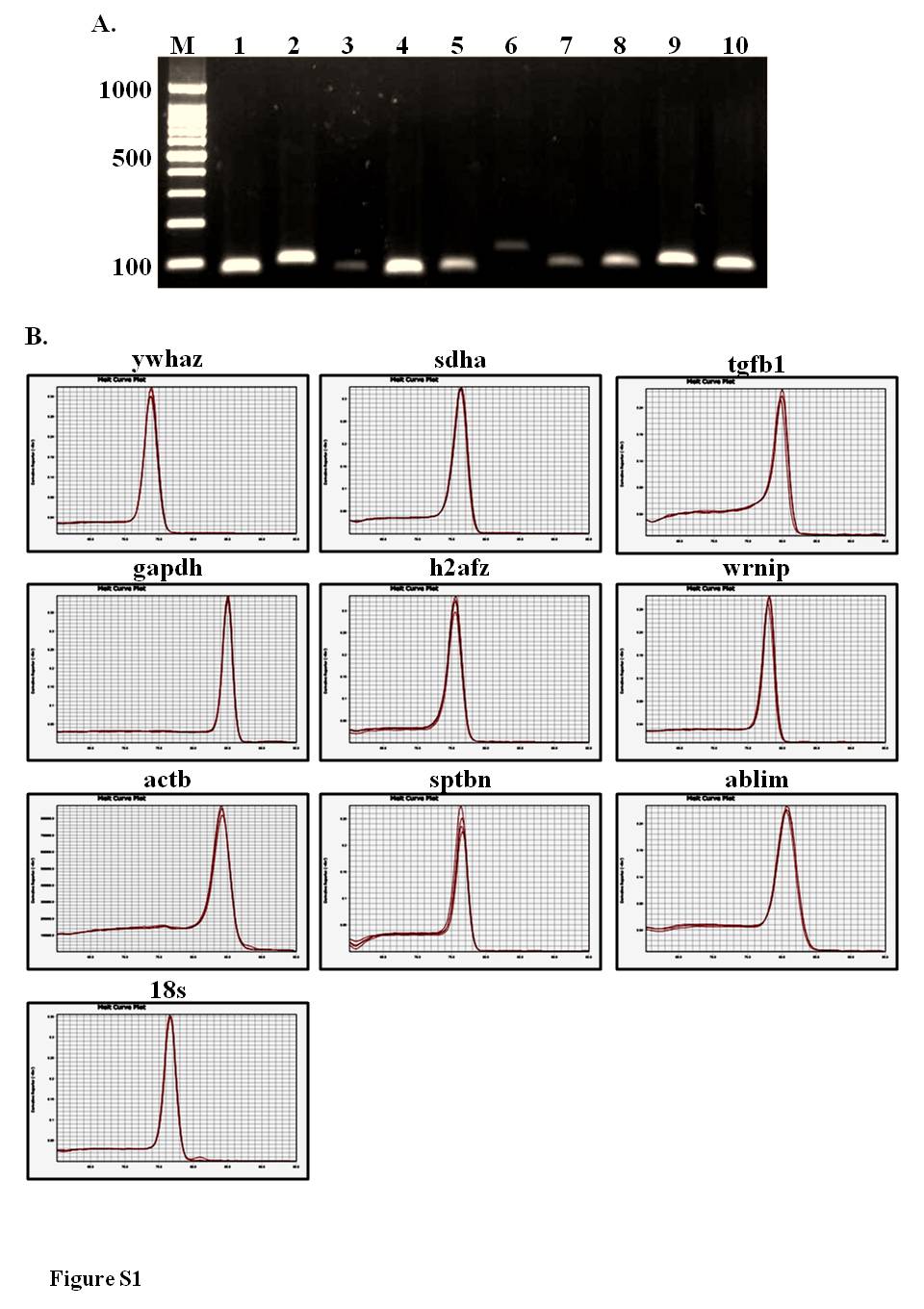

Supplement: Supplementary file 3 — Additional file 3: Figure S1: Selected reference gene specificity and amplification length. A. specific PCR product was analyzed on agarose gel (1.5%) electrophoresis for candidate housekeeping genes. Lane M : 100 bp DNA ladder marker. Lane 2 : ywhaz, Lane 3 : sdha, Lane 4 : tgfb1, Lane 5 : gapdh, Lane 6 : h2afz, Lane 7 : wrnip, Lane 8 : actb, Lane 9 : sptbn, Lane 10 : ablim, Lane 11 : 18s. B. Melting curve analysis of ten reference genes showing a single peak. (JPG 158 KB) [file 13104_2014_3197_MOESM3_ESM.jpg]

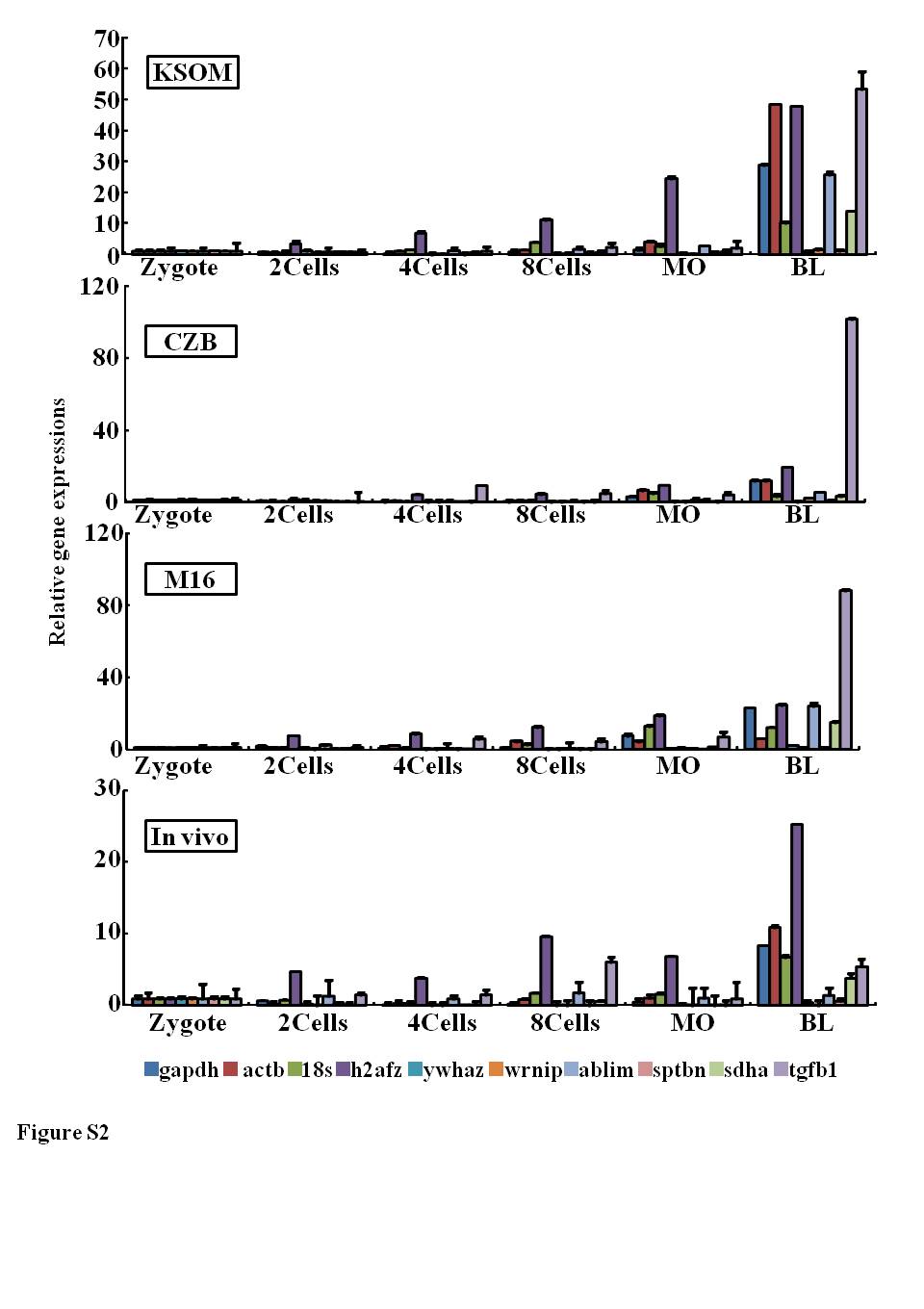

Supplement: Supplementary file 5 — Additional file 5: Figure S2: Selected reference gene expression levels of transcripts in the B6D2F-1 mouse-derived pre-implantation stage embryos. The transcript levels are shown for KSOM- (A), CZB- (B), M16- (C), and in vivo (D)-derived embryos. The expression at zygote stage was measured as a reference to calculate the relative amounts in the different stages. (JPG 89 KB) [file 13104_2014_3197_MOESM5_ESM.jpg]

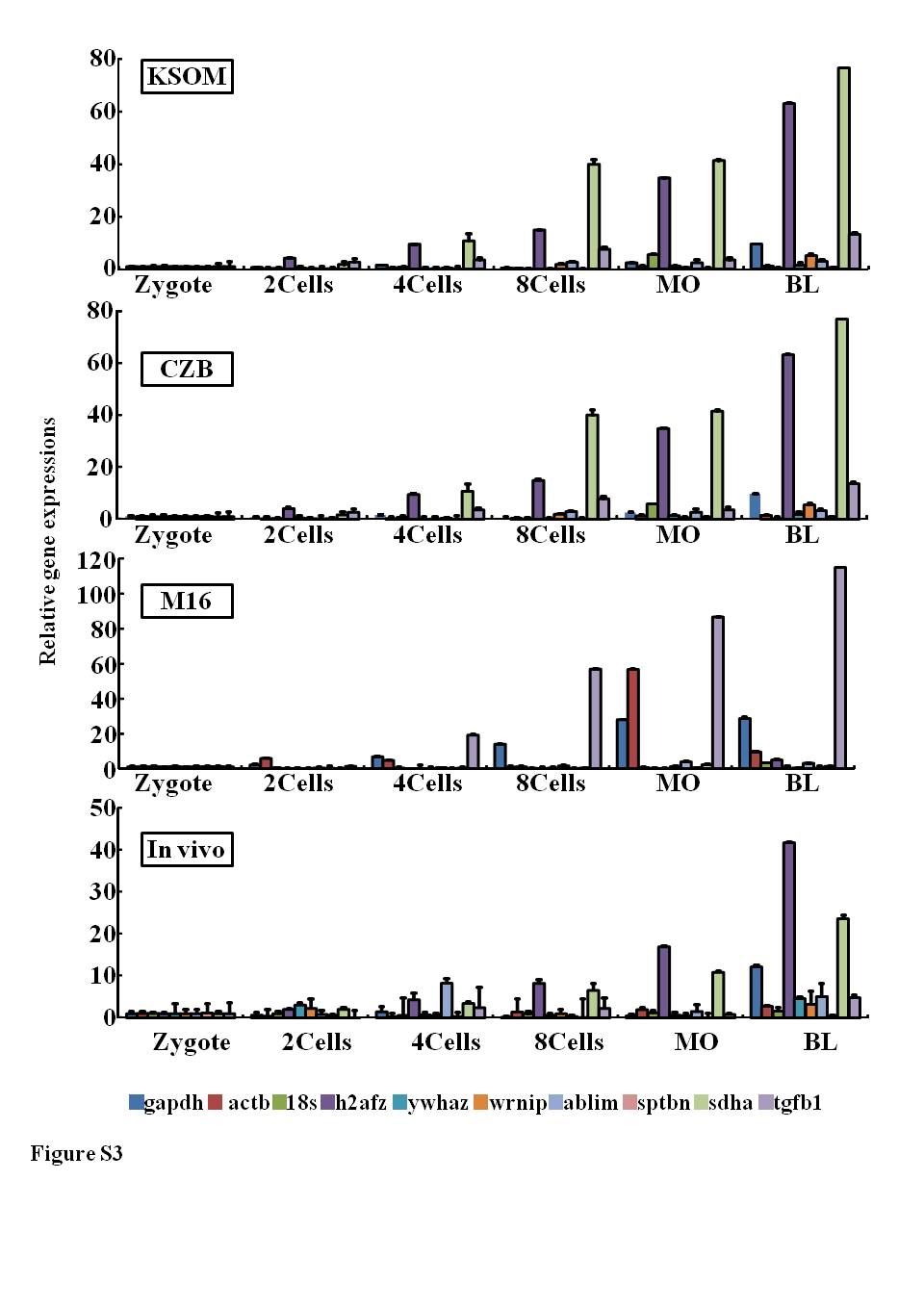

Supplement: Supplementary file 6 — Additional file 6: Figure S3: Candidate housekeeping gene expression levels of different transcripts detected in the C57BL/6 mouse-derived pre-implantation stage embryos. The expression levels of reference gene transcripts is shown for KSOM- (A), CZB- (B), M16- (C), and in vivo (D)-derived embryos. The expression at zygote stage was measured as a reference to calculate the relative amounts in the different stages. (JPG 96 KB) [file 13104_2014_3197_MOESM6_ESM.jpg]

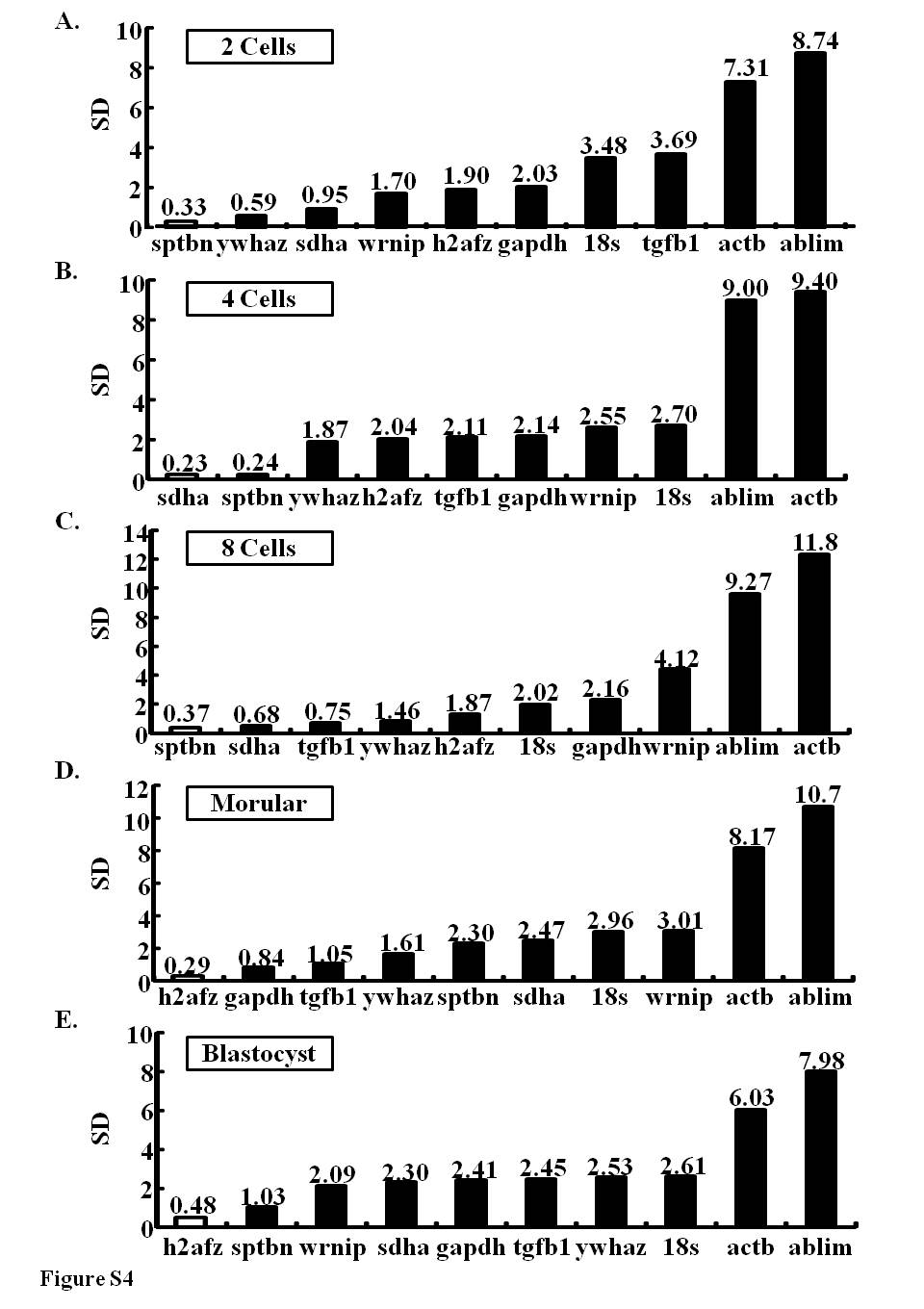

Supplement: Supplementary file 7 — Additional file 7: Figure S4: Rankings of selected housekeeping genes in in vivo-derived each stages embryos in B6D2F-1 mouse strains: A) 2-cell, B) 4-cell C) 8-cell D) morulae, and E) blastocyst stages. Data were obtained from A–E. Standard deviation (SD) of traditional housekeeping genes using NormFinder. The most stable genes are on the left and the least stable genes on the right. The experiments were performed in triplicate; data shown represent the mean of three independent experiments. (JPG 117 KB) [file 13104_2014_3197_MOESM7_ESM.jpg]

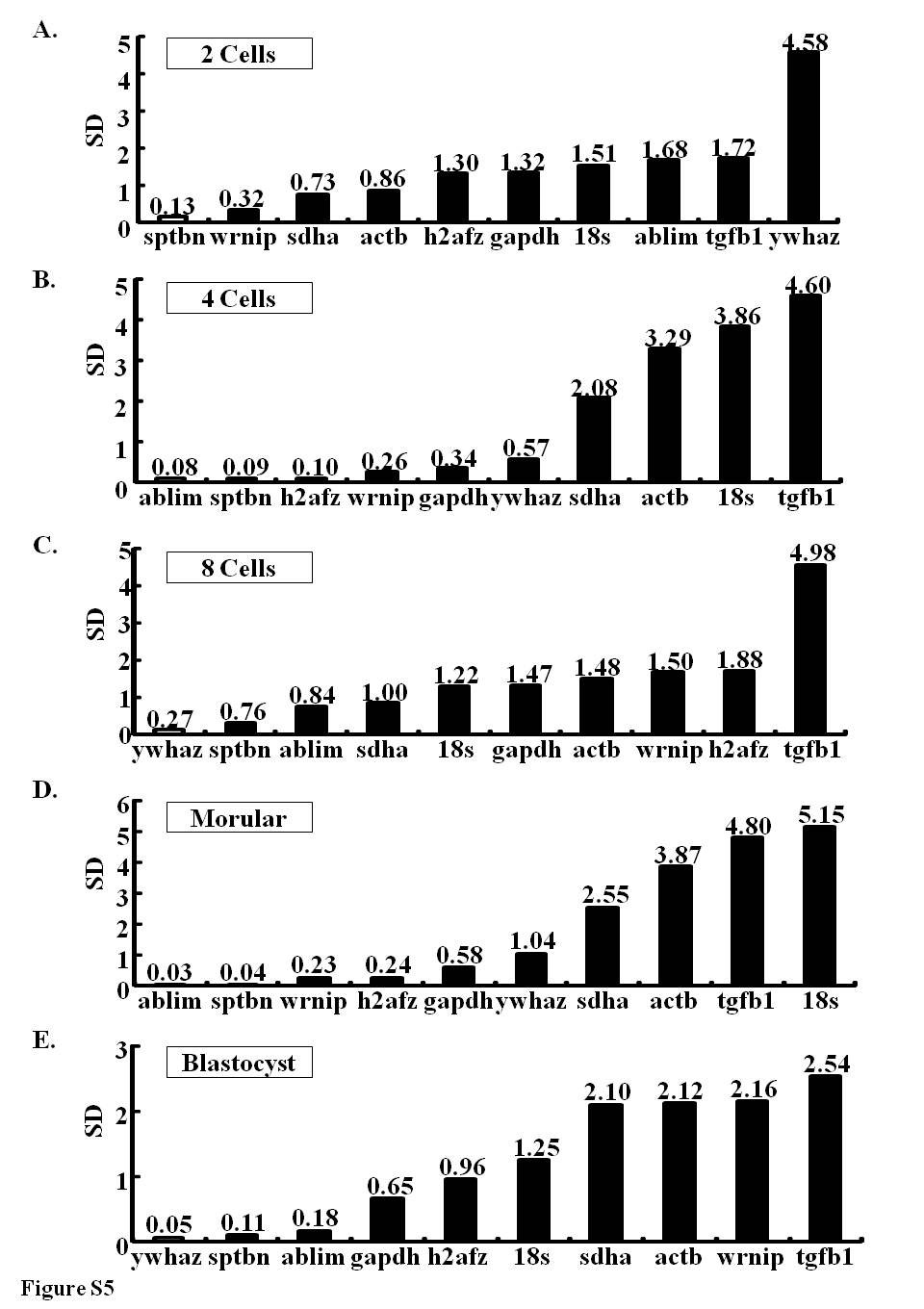

Supplement: Supplementary file 8 — Additional file 8: Figure S5: Average stability rankings of ten endogenous reference genes in each stages embryos in C57BL/6 mouse strains: 2-cell, B) 4- cell, C) 8-cell, D) morulae, and E) blastocyst stages. Data were obtained from A–E and analyzed for the SD of endogenous reference genes using the NormFinder program. The most stable genes are on the left and the least stable genes on the right. The experiments were performed in triplicate; data shown represent the mean of three independent experiments. (JPG 116 KB) [file 13104_2014_3197_MOESM8_ESM.jpg]

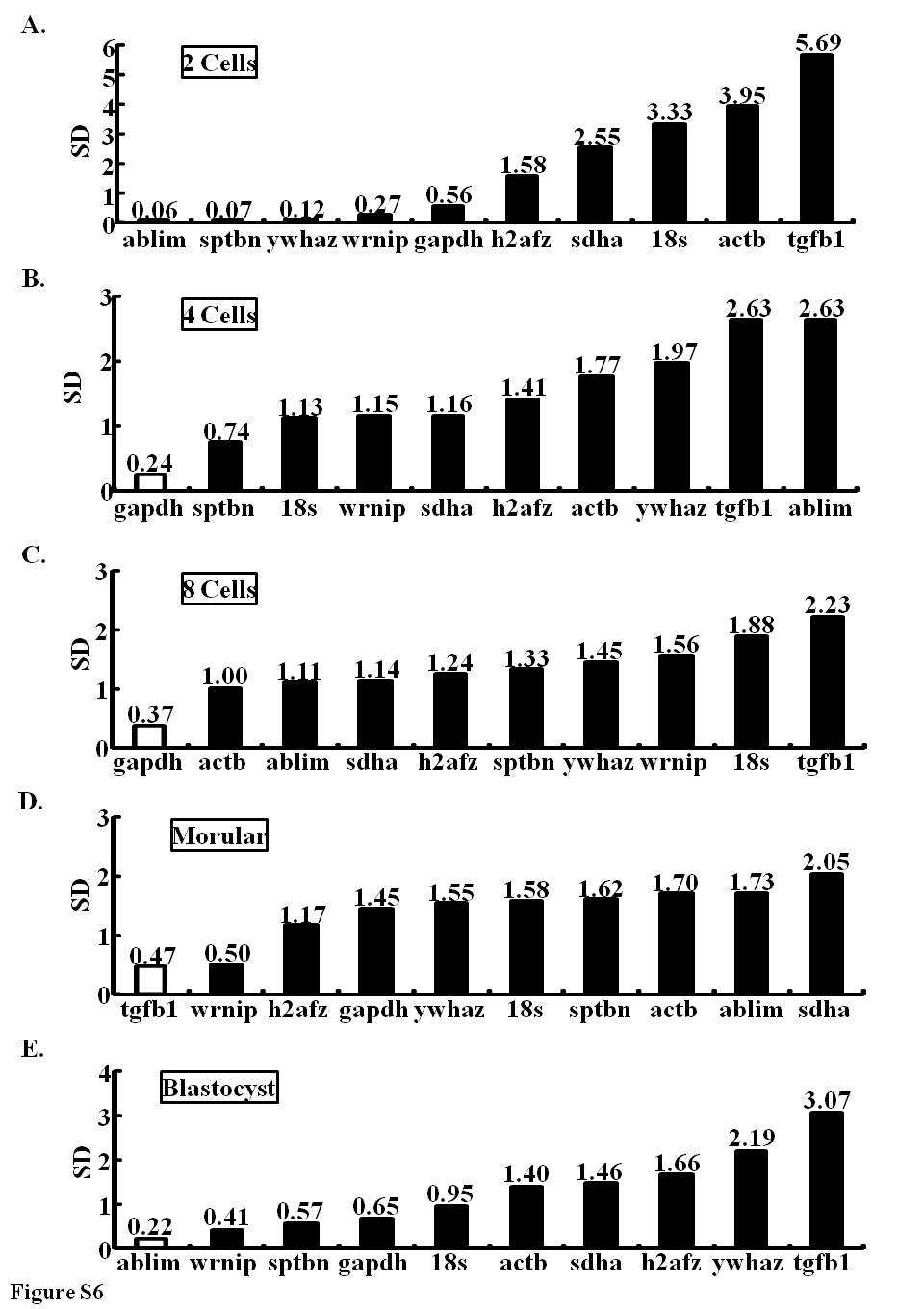

Supplement: Supplementary file 9 — Additional file 9: Figure S6: Rankings of ten housekeeping genes in in vivo-derived each stages embryos in ICR mouse strains: 2-cell, B) 4- cell, C) 8-cell, D) morulae, and E) blastocyst stages. Data were obtained from A–E, respectively. Ranking is based on the principle that gene pairs have stable expression patterns relative to each other and are considered appropriate housekeeping genes. The most stable genes are on the left and the least stable genes on the right. The experiments were performed in triplicate; data shown represent the mean of three independent experiments. (JPG 117 KB) [file 13104_2014_3197_MOESM9_ESM.jpg]
